# mPneumonia: Development of an Innovative mHealth Application for Diagnosing and Treating Childhood Pneumonia and Other Childhood Illnesses in Low-Resource Settings

**DOI:** 10.1371/journal.pone.0139625

**Published:** 2015-10-16

**Authors:** Amy Sarah Ginsburg, Jaclyn Delarosa, Waylon Brunette, Shahar Levari, Mitch Sundt, Clarice Larson, Charlotte Tawiah Agyemang, Sam Newton, Gaetano Borriello, Richard Anderson

**Affiliations:** 1 PATH, Seattle, Washington, United States of America; 2 Department of Computer Science and Engineering, University of Washington, Seattle, Washington, United States of America; 3 Kintampo Health Research Centre, Kintampo, Ghana; Georgia Institute of Technology, UNITED STATES

## Abstract

Pneumonia is the leading infectious cause of death in children worldwide. Each year, pneumonia kills an estimated 935,000 children under five years of age, with most of these deaths occurring in developing countries. The current approach for pneumonia diagnosis in low-resource settings—using the World Health Organization Integrated Management of Childhood Illness (IMCI) paper-based protocols and relying on a health care provider’s ability to manually count respiratory rate—has proven inadequate. Furthermore, hypoxemia—a diagnostic indicator of the presence and severity of pneumonia often associated with an increased risk of death—is not assessed because pulse oximetry is frequently not available in low-resource settings. In an effort to address childhood pneumonia mortality and improve frontline health care providers’ ability to diagnose, classify, and manage pneumonia and other childhood illnesses, PATH collaborated with the University of Washington to develop “mPneumonia,” an innovative mobile health application using an Android tablet. mPneumonia integrates a digital version of the IMCI algorithm with a software-based breath counter and a pediatric pulse oximeter. We conducted a design-stage usability field test of mPneumonia in Ghana, with the goal of creating a user-friendly diagnostic and management tool for childhood pneumonia and other childhood illnesses that would improve diagnostic accuracy and facilitate adherence by health care providers to established guidelines in low-resource settings. The results of the field test provided valuable information for understanding the usability and acceptability of mPneumonia among health care providers, and identifying approaches to iterate and improve. This critical feedback helped ascertain the common failure modes related to the user interface design, navigation, and accessibility of mPneumonia and the modifications required to improve user experience and create a tool aimed at decreasing mortality from pneumonia and other childhood illnesses in low-resource settings.

## Introduction

Pneumonia is the leading infectious cause of death among children less than five years of age [1]Estimates indicate that 935,000 children died from pneumonia in 2013—more than 2,500 children every day—accounting for 15 percent of child deaths globally. Childhood pneumonia can be difficult to diagnose, especially in low-resource settings (LRS) [2] Often pneumonia goes undiagnosed and untreated until the child is severely ill or is improperly managed by low-skilled health care providers (HCPs). Despite pneumonia-related mortality being preventable with simple interventions and appropriate treatment, diagnosing and managing childhood pneumonia in LRS remain challenging.

The current approach—using paper-based protocols and relying on an HCP’s ability to manually count breaths to calculate a respiratory rate (RR)—has proven inadequate in addressing this challenge [3–9] Recognizing difficult or fast breathing is central to the World Health Organization (WHO) Integrated Management of Childhood Illness (IMCI) and integrated Community Case Management algorithms, which are used to diagnose childhood pneumonia in LRS [10, 11] This is accomplished by visually observing the child’s breathing and measuring the RR, typically for one minute. However, assessing a child’s RR can be very challenging in a fast-breathing, moving child, and studies have shown that algorithms based on RR alone do not adequately identify children with pneumonia [2, 12–14] Furthermore, clinical assessments are not sufficient for determining the severity of the disease or the risk of complications. Pulse oximetry can noninvasively measure oxygen saturation and detect hypoxemia, a diagnostic indicator of the presence and severity of pneumonia often associated with an increased risk of death. Yet, hypoxemia is not routinely assessed in LRS because pulse oximetry is frequently not available [15] In addition, despite the WHO encouraging its use, pulse oximetry is not included in the current IMCI algorithm.

Recent evidence suggests that mobile health (mHealth)-based applications can improve HCP adherence to diagnostic and treatment guidelines [16–20] In an effort to address childhood pneumonia mortality and improve HCPs’ ability to diagnose, classify, and manage childhood pneumonia as well as other childhood illnesses, we developed “mPneumonia,” an innovative mHealth-based application using Android phone or tablet technology. mPneumonia integrates a digital version of the IMCI algorithm with a software-based breath counter to help HCPs more accurately count a child’s breaths and an off-the-shelf reusable pediatric pulse oximeter to detect hypoxemia; this provides a user-friendly diagnostic and management tool for childhood pneumonia and other childhood illnesses to improve diagnostic accuracy and facilitate adherence by HCPs to established diagnostic process. While pneumonia is a primary focus of this application given its large burden of disease among children, the mPneumonia application is not only intended for pneumonia diagnosis and treatment; it also facilitates the diagnosis and treatment of all childhood illnesses in accordance with IMCI.

## Methods

In collaboration with the University of Washington Department of Computer Science and Engineering, PATH employed an iterative development methodology to create and design the integrated mPneumonia prototype. We used Open Data Kit (ODK) Survey, a free open-source platform, to implement the complex and adaptable workflows of IMCI, provide interactive nonlinear navigation capabilities, and enable run-time customizable navigation and question data types [21]For example, ODK Survey was designed to accommodate questions with complex branching and user-directed nonlinear flow requirements. We used the ODK 2.0 Tools Suite to digitize and enhance the WHO IMCI diagnostic protocol, transforming the paper-based WHO IMCI algorithm into a step-by-step, user-friendly assessment questionnaire with embedded instructions, pictures, and videos on the Android operating system. The application did not require a network or wireless connection to function and could be used completely offline. It could store information locally until connected to a network, after which data files could be uploaded to a central server.

The software-based manual breath counter was developed to facilitate rapid identification of fast breathing, which was calculated and recorded by tapping the screen for each breath observed over a one-minute period. Visual, audio, and vibration indicators provided feedback to the user when a tap has occurred as well as when the measurement period has ended. The breath counter started as soon as the HCP began recording and stopped automatically when the measurement period was completed. The clinical algorithms were further adapted to interpret oxygen saturation data. The pulse oximeter data was recorded using an off-the-shelf Nonin 8000AP reusable pediatric finger clip pulse oximeter attached to a Nonin Xpod, enabling the connection between the pulse oximeter sensor and the mobile Android device. A custom device driver was written to integrate the Nonin pulse oximeter with the ODK Sensors framework. Nonin’s SmartPoint™ Algorithm was employed to indicate when the reading was high quality and the user should record the data (the numbers displayed on the Android device turned from pink to blue). This would prevent users from recording the pulse oximeter reading if the sensor was not worn correctly. While the pulse oximeter could accurately determine a child’s oxygen saturation, we chose not to use this feature in this study. Since we were only assessing the feasibility, usability, and acceptability of the mPneumonia application in this study, and not validating the application, breath counter, or pulse oximeter, we wanted to ensure the application was not used in the clinical care of the patient and that it did not influence the care of the patient in any way. Therefore, we set the oxygen saturation to read 99 percent at all times.

All components were integrated into a user-friendly decision support system, with clinical and physiological measurement results automatically guiding the protocol decision-making process and recommending the appropriate treatment or rapid referral to the closest facility with available treatments. The tablet language was in English, the preferred language among HCPs in Ghana. Requiring a username and password for access, mPneumonia was designed for secure use on Asus Nexus Google 7 tablets with a 7-inch portrait display running Android version 4.2.

mPneumonia is targeted for use by lesser-trained HCPs in LRS. In accordance with the consolidated criteria for reporting qualitative research, or COREQ, we undertook field usability testing in March 2014 among end-users in one district in the Brong-Ahafo region of Ghana [22] The objectives were to identify and resolve usability problems prior to a larger-scale introduction and pilot of mPneumonia within community health clinics in Ghana, and to increase the likelihood of successful integration into users’ daily surroundings and routines. The design and development process took into consideration local health policies, health system structures, and resource availability, which are especially important in LRS. After pilot testing the interview guides, purposive and snowball sampling techniques were used to recruit representative users from two peripheral health clinics. All HCPs that had knowledge about or provided direct treatment to children under five years of age were eligible. A tablet with the pulse oximeter hardware connected and software settings already preconfigured was provided to each HCP to use.

In a private setting at the clinic where the HCP worked, task analysis, concurrent think-aloud, and retrospective probing techniques were used to collect qualitative and quantitative usability metrics. Task analysis was used to understand in detail how the users interacted with mPneumonia. The researcher silently observed and focused on extracting the processes and tasks the users undertook when using the application. Concurrent with the task analysis exercise, the users were asked to “think aloud” and explain what they were doing, thinking, and feeling as they interacted with the application. The task analysis mapped the processes and steps the users performed, and this was supplemented by objective comments from the users as they verbalized their thought processes to yield information on the usability of mPneumonia. Retrospective probing was employed after the users completed their interactions with the application. The probing questions aimed to further examine user needs and perceptions, including perceived efficacy and usefulness of mPneumonia, and to get recommendations to improve the application. Thus, HCPs were asked to complete a case scenario without guidance or prior training in the use of mPneumonia, and to speak aloud while a researcher marked observations about user pathways and potential failure modes. A short questionnaire and system usability ratings were used to gather feedback around the perceived usability of mPneumonia.

Analysis of the data involved manually sorting and summarizing the data using a mixed methods semi-quantitative and qualitative approach guided by our evaluation criteria (Table 1). All data were cleaned, coded, and de-identified prior to analysis. The PATH research determination committee classified this as a design-stage evaluation activity. This human subject research was conducted according to the principles expressed in the Declaration of Helsinki, and written informed consent was obtained from all study participants.

**Table 1:** Evaluation Criteria for Initial Design-Stage Field Testing in Ghana.

Stage	Activity
**Exploratory**	Understand user workflow.
	Explore users’ work environments to determine if adjustments are necessary to make application easier to use.
	Investigate where questions or user confusion may arise when navigating the application.
	Obtain frontline HCPs’ initial impressions.
**User interaction**	Conduct tests to improve any protocol navigation or direction problems.
	Identify areas where the application is inefficient or displeasing to the user, and identify improvements that will make the user experience better.
	Investigate form factor and application robustness and ruggedness.
	Investigate how the user interacts with the breath counter and pulse oximeter.
**Workflow verification**	Verify application makes sense to users.
	Verify application works within field context.
	Investigate user confusion.
**Integrated testing**	Understand users’ perceptions of the application and determine areas of improvement for user comfort.
	Test battery life under normal working conditions.
	Anticipate and identify issues with application use during the pilot study, which will include formal acceptability and feasibility testing.
**Final verification**	Address any outstanding design issues or questions.
	Ensure application is ready for the pilot study.

## Results

### User demographics

Seven HCPs were enrolled in the design-stage usability evaluations in two peripheral government community health centers in the Nkoranza district of the Brong-Ahafo region in Ghana. Supervised by a midwife or community health officer (CHO) with the authority to prescribe medications, the health centers house a dispensary and basic laboratory. The IMCI-trained midwife or CHO oversees community health nurses (CHNs) who are also trained in IMCI to make referrals to the district hospital, which is staffed by physicians. Midwives, CHOs, and CHNs have completed at least two years of training. Nurses and field technicians with limited technical training provide additional health care support. All included HCP were literate and could read, write, and speak in English.

One midwife, one CHO, two CHNs, two enrolled nurses, and one field technician participated in the usability data collection and short interview. In order to maintain confidentiality, HCP responses are not reported by cadre here. The majority (five of seven) of these HCPs reported this experience to be their first time to use a touch-screen device and expressed the need to become familiar with the technology. Two HCPs owned standard mobile phones, two owned Android phones, and three did not own any mobile phones. Six of the seven HCPs were female. All seven HCPs were familiar with the IMCI assessment algorithm and medical terms presented within the application.

### Initial responses

Initial reactions to the mPneumonia application and tablet device were positive (Table 2). Most HCPs first commented on the attractive size and shape of the tablet. HCPs reported that the portable, “modern,” and slim design of the tablet was comfortable to hold and “easy…to use without glasses.” A few concerns regarding the robustness and reliability of the tablet and accessories, the need for regular maintenance and replacement parts, and potential theft or damage were raised. HCPs suggested that theft could easily be mitigated by providing a hard, lockable case to enable storage within a drawer at the clinic or at home. Most HCPs commented that the software appeared to be fast but found it difficult and struggled to get the application started.

**Table 2:** mPneumonia Prototype Initial Responses.

Survey item	Health care providers’ responses									
	A	B	C	D	E	F	G	Mean	Response	SD
**I am familiar with a tablet. This is a tablet.**	1	1	5	1	2	4	1	**2.1**	**Disagree**	1.7
**The shape of the tablet is appealing.**	5	5	5	5	5	5	5	**5.0**	**Strongly agree**	0.0
**The size of the tablet is just right.**	5	4	5	5	4	5	4	**4.6**	**Strongly agree**	0.5
**If it were available, I would like to use the tablet when I see pediatric patients.**	4	1	5	5	5	5	5	**4.3**	**Agree**	1.5
**The application looks easy to use.**	4	2	5	5	4	5	1	**3.7**	**Agree**	1.6

Note: 1–Strongly disagree; 2–Disagree; 3–Neither agree nor disagree; 4–Agree; 5–Strongly agree.

### Task analysis

All seven HCPs successfully completed the application procedures, corresponding with one patient encounter, within a mean of 43.4 (range 24 to 100) minutes. The task analysis activities identified 17 critical (made without noticing) and nine noncritical (almost made, or made and corrected) errors that prohibited HCPs from completing tasks within the application effectively and efficiently (Tables 3 and 4). Most critical errors were related to user interface design and software navigation buttons. Other critical failure modes were related to the first-time use of the breath counter and pulse oximeter, and responses to the IMCI assessment questions. If the HCPs misunderstood the IMCI assessment questions and submitted incorrect responses, mPneumonia would produce an incorrect classification. Noncritical errors were related to the tablet and hardware itself. Most HCPs were unfamiliar with touch-screen technology and thus were initially hesitant to use the device. HCPs struggled to identify how to change their answer selections but were later able to self-correct with practice. HCPs took the most time using the application during system navigation (when starting the tablet, using the touch screen, and using the QWERTY keyboard), software navigation (when opening and starting the application, moving between pages, reading instructions and IMCI text, and submitting answers), breath counter use, and pulse oximeter use. The HCPs failed to read the directions carefully and frequently attempted to skip questions; however, in an effort to increase adherence to the IMCI protocol, mPneumonia prohibits unanswered questions. The inclusion of images, such as for palmar pallor, seemed to enable HCPs to answer questions quickly without having to read the text.

**Table 3:** mPneumonia Prototype Task Analysis Summary: Critical Errors = Errors Made Without Noticing.

Error	Most frequently encountered issues	Changes made based on field test results
**Software navigation**	Identifying “Next” and “Back” buttons. Pressing hardware instead of software buttons.	Added “Next” and “Back” buttons to bottom of navigation bar. Included small icon in addition to text.
	Recognizing and utilizing software buttons.	Included instructions and labels such as “Press here” for navigation buttons and “Not specified” in text boxes with unanswered responses. Removed use of boxes and underlines to outline important instructions and replaced with alternate colors and bold text. Enlarged buttons and included color.
	Using table of contents button.	Relabeled to “Menu.”
	Recognizing completion progress in application.	Inserted major IMCI section headings.
**Survey design**	Selecting all responses that apply instead of single answer.	Reformatted question to: “Does the child have any of the following?” Inserted red color change to signal answer selection and provide feedback to user.
	Selecting “Present” instead of “Not present.”	Reformatted question to simple yes/no questions.
	Selecting and submitting answers (e.g., buttons spaced too closely to each other, pressed “no” without realizing it because no feedback provided with answer selection).	Changed arrangement of answer buttons from vertical to horizontal. Resized buttons. Inserted red color change to signal answer selection and provide feedback to user.
	Submitting birth date.	Resized date widget. Changed from MM/DD/YYYY to DD/MM/YYYY format.
	Recognizing whether question is directed toward health care provider or caregiver.	Inserted prompts “Health care provider looks” and “Ask the caregiver” above questions.
**IMCI content**	Understanding IMCI questions (e.g., “Is the child not able to drink or breastfeed?" Yes/no double negative).	Inserted red color change to signal answer selection and provide feedback to user.
	Understanding IMCI terminology, disease processes, and subject matter (e.g., concept of palmar pallor).	Included images where possible.
	Answering questions if not applicable to setting or resources not available.	Inserted “Not applicable” or “Not available” answer selections.
	Interpreting results (e.g., if child had two illnesses or required two treatments).	Inserted summary of symptoms selected that contributed to classification. Inserted explanations such as “Give the child amoxicillin for the treatment of pneumonia” to provide feedback to user.
**Breath counter**	Launching breath counter.	Included instructions such as “Press here to start.” Enlarged buttons and included color.
	Using breath counter (e.g., not tapping screen for every breath observed, not appreciating when the counter started).	Inserted emphasis in instructions to tap for each breath: “Press here for EACH breath.” Enlarged buttons and included color.
	Understanding results after one minute.	Inserted instructions with description. Inserted table with World Health Organization RR cutoff rates for age. Included interpretation of results and feedback to user based on breath count and age (e.g., automatically calculates if fast breathing is present: green—child’s RR is normal for age; red—child has fast breathing > 50 breaths per minute).
	Recording results.	Inserted emphasis on instructions to “Press here to submit results.” Enlarged buttons and included color.

Abbreviations: DD = day; IMCI = Integrated Management of Childhood Illness; MM = month; RR = respiratory rate; YYYY = year.

**Table 4:** mPneumonia Prototype Task Analysis Summary: Noncritical Errors = Errors Almost Made, or Made and Corrected Appropriately.

Error	Most frequently encountered issues	Changes made based on field test results
**Tablet**	Turning on tablet.	Inserted green stickers to label power button on tablet.
	Applying sufficient pressure to touch screen.	Training and practice.
	Minimizing pop-up keyboard.	Instructed user to press “enter” on keyboard to submit answer and remove keyboard.
**Pulse oximeter**	Positioning fingertip probe.	Inserted instructions with image and description.
	Launching pulse oximeter.	Included instructions such as “Press here to start.” Enlarged buttons and included color.
	Recording results.	Inserted emphasis on instructions to “Press here to submit results.” Enlarged buttons and included color.
	Completing measurement and removing probe from finger.	Inserted sound to indicate to user when to record pulse oximetry reading. Inserted instructions to remove probe from finger after reading recorded.
**Error recovery**	Changing answer selections.	Enabled red color change to signal answer selection to user.
	Typing errors	Training and practice.

### Ease of use and learnability

All seven HCPs reported that they imagined that most people would learn to use the application quickly. HCPs using a touch screen for the first time noted the need to become familiar with the technology and software. One user explained this commonly expressed sentiment by saying, “It wasn’t easy because I was not conversant with the keys. Frequent use of the device will help a lot.” HCPs who did own an Android phone demonstrated the ability to move through the application relatively quickly. Short interviews revealed that all HCPs expressed a desire and willingness to use the application and thought nominal training and technical support would be required for first-time users. HCPs reported that an average of 16.3 hours, or two days, of training would be required to become fully comfortable using the application with pediatric patients.

### Content accuracy and appropriateness

HCPs provided their comments on the accuracy of the clinical information in the mPneumonia application and appropriateness at their level of the health system. HCPs expressed appreciation for having all of the assessment questions available in sequential order and predicted that use of mPneumonia would reduce the amount of paperwork compared to the paper-based protocol. Overall, all HCPs preferred mPneumonia to the paper-based protocol. HCPs commented that mPneumonia would provide a more accurate and automatic classification or referral decision. HCPs requested that additional detailed medical advice (e.g., predisposing factors for pneumonia, feeding assessments, and counseling information for mothers and children being referred) be included, as well as all diagnoses for use with all patient populations and conditions. Although researchers identified during the task analysis that HCPs struggled to use the breath counter and pulse oximeter the first time, the questionnaire revealed that HCPs found both of these tools to be easy to use and helpful for their work. One user summarized, “It would make our work easier and fast for treatment or referrals. It is easy to me because you will not have to manually count anything.”

Further analysis of the qualitative data revealed clinical and technical experience, resources, and context largely defined user expectations and behaviors. HCPs with minimal training, experience, or lack of adherence to IMCI guidelines often expressed surprise or confusion with the IMCI classification results. However, HCPs with greater levels of training and experience confirmed agreement with the IMCI protocol of the mPneumonia application. Study HCPs remarked that mPneumonia could add value to the management of childhood pneumonia by increasing patient demand and efficiency of clinic visits.

### User satisfaction

Initially hesitant, HCPs complained about the lack of instruction prior to use of mPneumonia and demonstrated difficulties during their first-time use. However, by the end of their user experiences, all seven HCPs expressed overall high levels of satisfaction and that they would use the application frequently if it were available (Table 5). HCPs also suggested that mPneumonia could be useful to those HCPs outside of the clinic who perform referrals and community outreach.

**Table 5:** mPneumonia Prototype Systems Usability Scale Survey Responses.

Survey item	Health care providers’ responses									
	A	B	C	D	E	F	G	Mean	Response	SD
**I think that I would like to use this application frequently.**	5	5	5	4	5	5	5	**4.9**	**Strongly agree**	0.4
**I found this application unnecessarily complex.**	2	4	1	1	1	1	4	**2.0**	**Disagree**	1.4
**I thought the application was easy to use.**	2	3	5	4	3	2	4	**3.4**	**Neither agree nor disagree**	1.1
**I think that I would need the support of a technical person to be able to use this application.**	2	1	2	4	5	4	5	**3.3**	**Neither agree nor disagree**	1.6
**I found that the various steps in this application were well integrated.**	4	5	4	5	5	5	4	**4.6**	**Strongly agree**	0.5
**I thought that there was too much variation in this application.**	2	5	2	2	2	1	4	**2.6**	**Neither agree nor disagree**	1.4
**I would imagine that most people would learn to use this very quickly.**	4	5	5	5	5	5	5	**4.9**	**Strongly agree**	0.4
**I found the tablet and application very awkward to use.**	2	5	1	1	1	2	1	**1.9**	**Disagree**	1.5
**I feel very confident using the application.**	4	5	5	4	5	2	4	**4.1**	**Agree**	1.1
**I needed to learn a lot of things before I could get going with this application.**	4	4	1	2	2	3	2	**2.7**	**Neither agree nor disagree**	1.3

Note: 1–Strongly disagree; 2–Disagree; 3–Neither agree nor disagree; 4–Agree; 5–Strongly agree.

Abbreviations: SD = standard deviation.

## Discussion

The growing success of mHealth applications in LRS demonstrates the potential for a childhood pneumonia application to transform health care service delivery and improve health outcomes. Recent evidence suggests that digital technology and mHealth-based algorithms can enhance the ability of peripheral and lower-level facility and community HCPs to follow diagnostic and treatment guidelines [9, 16–20, 23] Our approach is to expand the technology options for assessment, classification, and management of pneumonia and other childhood illnesses through the development of an innovative mHealth application that integrates IMCI and a diagnostic and clinical decision support algorithm with a breath counter and a low-cost portable pulse oximeter.

Current standards of care are critically dependent upon the correct application of the WHO IMCI and integrated Community Case Management protocols for identification and treatment of childhood illnesses. However, use of the WHO protocols remains limited due to a lack of trained HCPs, insufficient supervision, and inadequate financial resources [3, 5–7] Even with training, HCPs often do not adhere to the WHO guidelines in practice, leaving clinical assessments incomplete and children requiring urgent referrals missed [6, 24] mPneumonia is designed to minimize the need for training, improve adherence to the IMCI algorithm, and provide clear guidance for interpreting results. The step-by-step software increases adherence to the algorithm by not allowing HCPs to skip essential assessment questions; this enables lesser-trained HCPs to identify children with pneumonia and other childhood illnesses and, most importantly, those at highest risk of death and in need of immediate treatment.

Measuring a child’s breath rate through visual observation is challenging, requiring focused concentration. Inaccurate or imprecise measurements can stem from difficulty counting or remembering the count [12] The mPneumonia breath counter enables the HCP to focus exclusively on observing and recording each breath without also needing to keep track of the count or the time. mPneumonia includes a pulse oximeter. While pulse oximetry is a critical component in the management of severe pneumonia and its use encouraged by the WHO, there is no designated step for it in the current IMCI algorithm and it is frequently not available in LRS. By providing a quantitative indicator, mPneumonia allows for more effective and cost-effective use of oxygen, a lifesaving therapy [25] Additionally, pulse oximetry can be beneficial even in the absence of supplemental oxygen because it can be used to prioritize or urgently refer patients to facilities with more intensive care[26].

**Fig 1:**
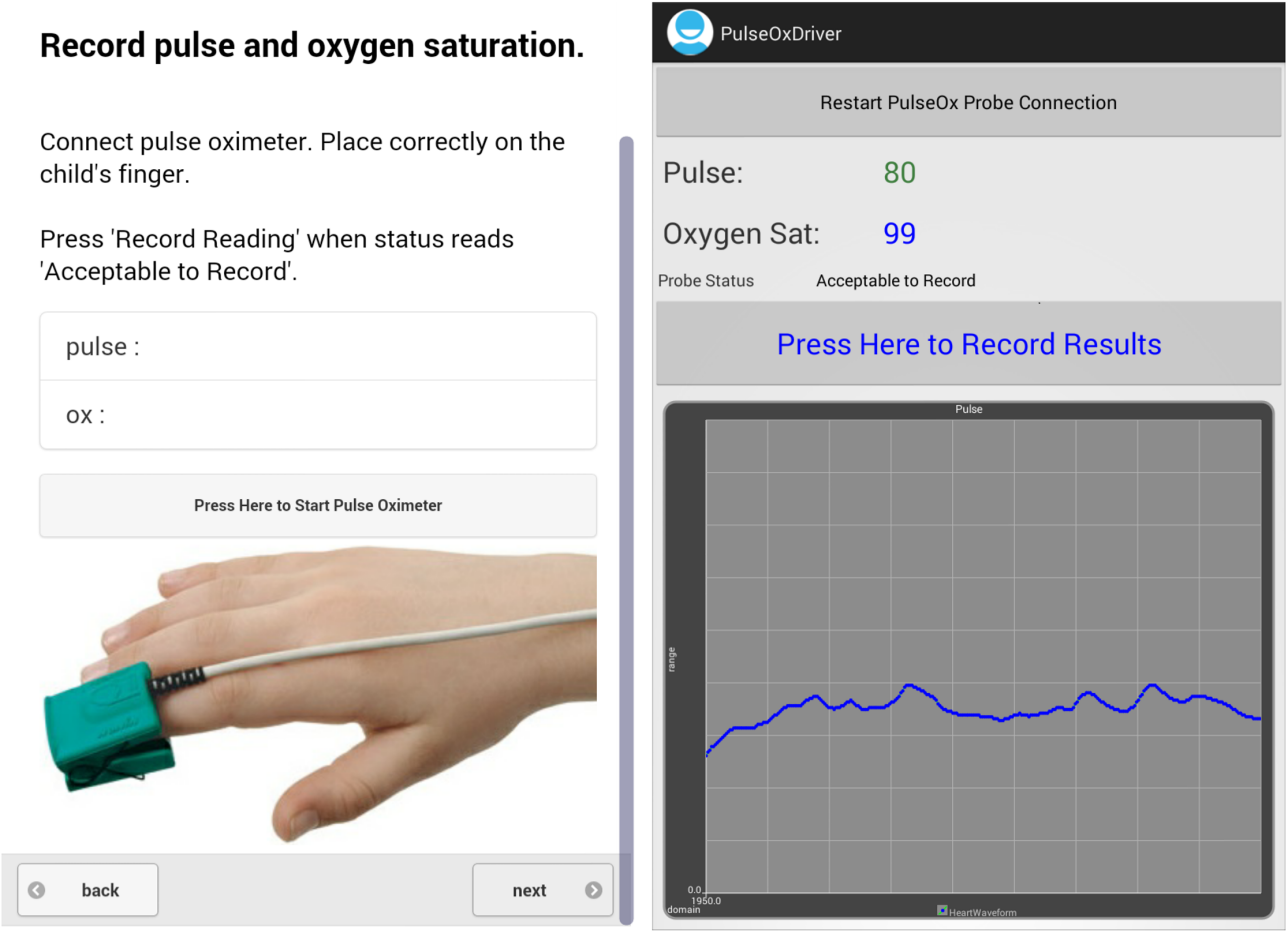
Screenshots Displaying mPneumonia Assessment Questions and Pulse Oximeter.

The results of the initial field test in Ghana provided valuable information for understanding the usability and acceptability of mPneumonia among HCPs. This critical feedback helped identify the common failure modes related to the user interface design, navigation, and accessibility of mPneumonia and the modifications required to improve the user experience (Tables 3 and 4). Larger-sized buttons and the use of colors have now been incorporated into the next iteration of the mPneumonia prototype to address the frequent misinterpretation of the software buttons as a set of instructions instead of as a key to press. Additional changes included: removing scrolling, enlarging font size of text, simplifying questions to “yes/no” answers, providing feedback to the HCP on answer selections, positioning answer selection buttons horizontally instead of vertically, and adding navigation buttons to the bottom of the screen (Fig 1). Appropriate utilization of different colors, shapes, sizes, shading, icons, and images may improve the user experience and have the potential to translate into significant improvements in the efficiency and accuracy of mPneumonia.

**Fig 2:**
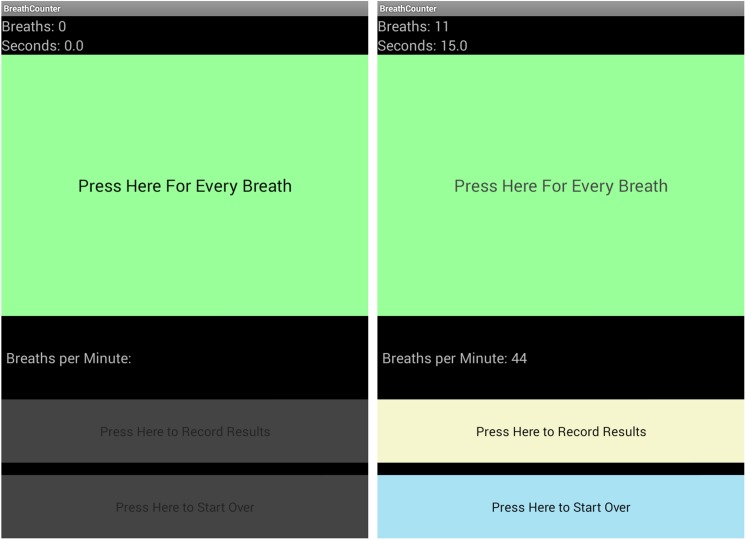
Screenshots Displaying mPneumonia Breath Counter.

User expertise and experience were influential factors, determining preferences for customization of the navigation and content of mPneumonia. Decreasing the amount of text necessary for the HCP to read and inserting symbols and images in the application (e.g., how the pulse oximeter probe should be placed on the finger) can reduce confusion and facilitate easier and faster use (Fig 2). Possibly burdened by high patient workloads, HCPs frequently tried to skip assessment questions. Additional error messages have been inserted into the next iteration to prevent HCPs from accidentally jumping ahead in the IMCI protocol. Given that clinicians average 8.2 minutes per child for a consultation using the paper-based IMCI protocol, 43 minutes (average time to complete the task analysis) is likely not feasible for most HCPs [27] It will be important to monitor how long it takes HCPs to navigate the application with daily routine use and increased familiarity.

Overall, the initial field test revealed that HCPs viewed mPneumonia favorably. HCP were in agreement on the choice of the tablet rather than an Android phone. HCPs preferred the large size and shape of the tablet. The tablet also reduced the risk of HCPs inserting their SIM cards and using the device for personal use. The capability of mPneumonia to operate completely offline not only provided significant benefit for health clinics lacking connectivity, it also inadvertently promoted the restricted use of the tablet for patient care. Despite concerns expressed by HCPs regarding theft, damage, and lack of access to electricity, all HCPs commented on the perceived value of mPneumonia and expressed interest in using mPneumonia if it were available.

The mPneumonia application has limitations, including the language of IMCI questions and the tablet itself. To address these limitations for the planned pilot study, we did not change the text of the IMCI questions but instead addressed confusion related to double negative IMCI questions by altering the user interface design and inserting red color feedback to highlight answer selections correlating with an abnormal exam finding. We developed a pocketbook mPneumonia user manual, and will provide training and an opportunity to practice using the mPneumonia application during the pilot study. For example, we will try to address in training the basics of using a QWERTY keyboard and that the user must press “enter” on the keyboard when submitting a response to make the pop-up keyboard disappear. There was also limited ability to modify the structure of the ODK Survey platform to adapt it to context-specific needs of user populations. To accommodate varying levels of training and experience among HCPs and resource availability across clinics, we identified the need to improve the flexibility of the system and display format to permit the complex alignment of the content and sequence of mPneumonia with local Ghana IMCI protocols and clinical practice. Despite these limitations, ODK Survey provides a free, open-source, simple and usable platform for designing and implementing IMCI protocols. With additional feedback from HCPs on how to increase the usability of the platform, the functionality of the ODK Survey platform has the potential to expand and improve.

Recommendations for future iterations to expand the functionality of mPneumonia included adding and synchronizing patient and drug inventory registries as well as providing educational support. A patient registry could enable quick access to the child’s vaccination records and monitoring of the child’s growth over time. Since clinics varied in their availability of drug options, a drug inventory registry could allow the flexibility to select the appropriate treatments based on availability. A drug inventory registry allowing the user the ability to pre-select and maintain the drugs available in the clinic could also provide restock reminders when drugs are running low. Another suggestion was a tool tip pop-up window allowing the HCP to scroll onto an unfamiliar term for additional information (e.g., images or videos). Future pre- and post-evaluation studies are needed to assess HCPs’ knowledge and adherence to the IMCI guidelines before and after use of mPneumonia to validate whether mPneumonia is a useful tool to facilitate increased adherence to IMCI guidelines. Furthermore, the clinical effectiveness of mPneumonia still requires investigation, as does determining the sustainable uptake. Research regarding the cost-effectiveness of mPneumonia is also needed. A costing study was outside the scope of this field test, and most of the components of the mPneumonia application were donated. However, since ODK Survey and the breath counter application are freely available Android applications that run on commodity Android devices, the main driver of cost of mPneumonia is likely to be the Android-compatible pediatric pulse oximeter.

This study provides a model for assessing the usability and acceptability of an mHealth application among HCPs, and identifying approaches to iterate and improve. The areas for improvement identified during this design-stage field test were essential to ensure that the application was ready for the pilot study. The field testing allowed PATH to provide a set of recommendations to the technology developers that translated directly to the iterative design, improved interface, and enhanced functionality of an adapted mPneumonia. The implemented changes will be tested during a pilot study of the acceptability and feasibility of introducing mPneumonia among HCPs and caregivers of children in community health clinics in the Kintampo North and South districts of Ghana.

## Conclusion

mPneumonia integrates an easy-to-use, step-by-step guide to improve adherence to diagnostic protocols, incorporates physiological measurements such as RR and pulse oximetry, and provides overall decision-making support. Accelerating the development of new childhood pneumonia innovations and spurring the adaptation of current mHealth devices could revolutionize pneumonia diagnosis and treatment. It will be important to prioritize the needs of HCPs and the constraints of using this technology in a LRS. New innovations must not only be evaluated for their effectiveness in diagnosing and treating children but also must be assessed for use in LRS. mPneumonia has the potential to enable rapid diagnosis and assessment, reduce the risk of death due to delayed treatment, and make an impact on the leading causes of childhood mortality.

## Supporting Information

S1 Table Dataset. (XLSX)
